# Costs of mass drug administration for scabies in Fiji

**DOI:** 10.1371/journal.pntd.0010147

**Published:** 2022-02-03

**Authors:** Maria Mow, Li Jun Thean, Matthew Parnaby, Jyotishna Mani, Eric Rafai, Aalisha Sahukhan, Mike Kama, Meciusela Tuicakau, Joseph Kado, Lucia Romani, Daniel Engelman, Margot Whitfeld, John Kaldor, Andrew Steer, Natalie Carvalho

**Affiliations:** 1 Tropical Diseases Group, Murdoch Children’s Research Institute, Melbourne, Victoria, Australia; 2 Department of Paediatrics, University of Melbourne, Melbourne, Victoria, Australia; 3 Ministry of Health and Medical Services, Suva, Fiji; 4 Wesfarmers Centre for Vaccines and Infectious Diseases, Telethon Kids Institute, Perth, Western Australia, Australia; 5 Kirby Institute, University of New South Wales, Sydney, New South Wales, Australia; 6 Melbourne Children’s Global Health, Melbourne Children’s Campus, The Royal Children’s Hospital, Melbourne, Victoria, Australia; 7 Department of Dermatology, St. Vincent’s Hospital, Sydney, New South Wales, Australia; 8 School of Medicine, University of New South Wales, Sydney, New South Wales, Australia; 9 School of Population and Global Health, University of Melbourne, Melbourne, Victoria, Australia; Imperial College London, UNITED KINGDOM

## Abstract

In 2019, the Murdoch Children’s Research Institute in partnership with the Fiji Ministry of Health and Medical Services carried out an integrated mass drug administration (MDA) for the treatment of scabies and lymphatic filariasis in the Northern Division of Fiji (population estimate 131,914). We conducted a retrospective micro-costing exercise focused on the cost of scabies control in order to inform budgeting and policy decision making in an endemic setting. We collected detailed information on financial and economic costs incurred by both parties during the course of the MDA campaign (April 2018 to July 2019). We also conducted interviews with personnel involved in the financial administration of the MDA campaign. The economic cost of delivering two doses of ivermectin was US$4.88 per person. The cost of donated drugs accounted for 36.3% of total MDA costs. In this first large-scale MDA for the public health control of scabies, the estimated cost of delivering MDA per person for scabies was considerably more expensive than the costs reported for other neglected tropical diseases. The important cost drivers included the remuneration of health care workers who were extensively involved in the campaign, coverage of hard-to-reach, mainly rural populations and the two-dose regimen of ivermectin. These results highlight the importance of these cost determinants and can be used to plan current and future MDA programs.

## Author summary

Scabies poses a significant burden on both health and economic systems. The Global Burden of Disease estimated that this skin disease affects more than 200 million people globally. However, the economic burden of scabies has not been studied widely and there are limited data on the cost of treating scabies in highly endemic areas. We conducted a costing study of a mass drug administration (MDA) program in the Northern Division of Fiji (population of 131,914). We collected financial and economic costs of administering ivermectin and permethrin to the whole of the Northern Division population in order to estimate the per capita cost of delivering MDA. The costs of MDA for scabies in Fiji were higher than those estimated in previous studies of MDA for other neglected tropical diseases. The study highlights significant cost components of implementing a large-scale MDA and provides key insights for the design and implementation of future MDA programs for scabies. It also adds to the general knowledge in understanding cost inputs and estimating the overall cost-effectiveness of this public health intervention.

## Introduction

Scabies is a neglected tropical disease (NTD) caused by the microscopic mite *Sarcoptes scabiei* var. *hominis*, and is estimated to affect more than 200 million people globally.[1] The mite burrows into the skin and lays eggs, and infestation leads to intense itching. The scratching of skin lesions breaks the skin barrier and can lead to bacterial skin infections such as impetigo, abscesses and cellulitis. Skin and soft tissue infections can in turn lead to more severe complications such as septicemia, kidney disease and possibly, rheumatic heart disease.[2] The economic burden on national health systems due to managing the symptoms of scabies and its secondary complications, along with the impact on productivity as a result of school and work disruptions are considerable.[3–4] Substantial economic costs have been associated with managing scabies outbreaks in several settings.[5–6] Furthermore, individuals affected by scabies can experience social stigma with feelings of shame and embarrassment.[7].

The 2015 Global Burden of Disease study found scabies to be more prevalent in tropical countries, with Fiji listed among five countries with the greatest burden of disease.[1].

The Skin Health Intervention Fiji Trial (SHIFT) study, conducted during 2012–13 in six Fijian island communities, observed an all-ages prevalence of scabies of 36.4%, with highest prevalence in children aged 5 to 9 years at 55.7%.[2] In an earlier population-based study conducted in 2007, the national prevalence of scabies in Fiji was estimated at 23.6% with the Northern Division recording the highest at 28.5%.[8].

In its latest roadmap for NTDs (2021 to 2030), the World Health Organization (WHO) identified mass drug administration (MDA) as a key public health intervention to address multiple NTDs, including scabies.[9] In Fiji, a nationwide MDA for scabies is currently being planned as part of the World Scabies Program(WSP).[10] MDA has been found to be effective in eliminating NTDs with high prevalence if implemented alongside additional public health measures.[11] For example, in the WHO Western Pacific region, 12 out of 24 endemic countries were certified for the elimination of lymphatic filariasis following annual MDA programs.[12].

A number of studies have demonstrated the effectiveness of ivermectin-based MDA for the treatment and control of scabies.[13–14] In Fiji, SHIFT demonstrated a decline in scabies prevalence of 94%, twelve months after ivermectin-based MDA.

According to the WHO, each US$1 invested in MDA programs for five NTDs (namely, lymphatic filariasis, onchocerciasis, schistosomiasis, soil-transmitted helminthiases and trachoma) is expected to yield an estimated US$25 net benefit to the individual, averting out-of-pocket spending on health and costs in lost productivity.[9] While there is some evidence that MDA programs are good value for money, there is still limited evidence on the cost of these programs. Existing MDA costing studies have primarily been conducted in African and Asian countries, with few, if any, conducted in the Pacific region, or in upper-middle income countries (LMIC), and no costing studies specific to scabies.[15–19] In earlier MDA programs targeting school aged children for soil transmitted helminth treatment programs, unit costs of less than one US dollar were reported.[16] Costing exercises for malaria MDA programs presented varied results, with the highest unit delivery cost recorded of US$11.05 in Comoros and the lowest cost recorded of US$0.36 in Sierra Leone.[19] In a recent study of MDA for malaria in Myanmar, the cost of delivering three rounds of targeted MDA was estimated at US$2.50 per person reached.[17] Given the wide variation in reported unit costs in the literature, policy-makers in low-to-middle income countries with constrained budgets require country- and context-specific costing estimates for the prioritisation of public health interventions and for planning and forecasting of health budgets.

The objective of this study was to estimate the total programmatic cost and unit cost of the Big SHIFT MDA from a health payer perspective. Big SHIFT was a study using ivermectin-based MDA for scabies control to determine its effect on the serious bacterial complications of scabies. The trial delivered its intervention to the population of Fiji’s Northern Division (estimated at 132,000).[20] These estimates can be used in future cost-effectiveness studies of MDA for scabies in Fiji and elsewhere in the Pacific where the context is similar. This evidence is also important for local and international policy-makers for planning and forecasting of health budgets in the context of the WSP, the first global program dedicated to the elimination of scabies as a public health problem.[10].

## Methods

### Ethics statement

This costing study formed a part of the overall Big SHIFT project and approval was granted by the Fiji National Health Research Ethics Review Committee (reference number 2018.38.NOR) and the Royal Children’s Hospital Human Research Ethics Committee, Melbourne, Australia (reference number 38020).

### Study setting and intervention

In this study, we collected and analysed cost data from Big SHIFT. While Big SHIFT was focused on MDA for scabies control, during the lead-up to the study in 2018, a transmission assessment survey revealed the prevalence of lymphatic filariasis in the Northern Division was above 1%, necessitating another MDA for control of lymphatic filariasis.[21] The campaign therefore also aimed to deliver triple therapy for control of lymphatic filariasis consisting of one dose of ivermectin, diethylcarbamazine (DEC) and albendazole simultaneously during the first MDA round.[22] The addition of a second dose of ivermectin and provision of permethrin for scabies ensured an integrated MDA approach; delivered 7 to 14 days after the first round. Permethrin 5% topical cream was offered in place of ivermectin to pregnant women, mothers breastfeeding infants less than a week old, children less than 90cm in height and patients on warfarin treatment. A height dosing pole was used to estimate the dose of ivermectin (S1 Table)[23] and administered using directly observed therapy (DOT).

There were three distinct phases of the campaign that included the start-up phase up as well as implementation of drug delivery activities, covering a period of 16 months from April 2018 through to July 2019. Advocacy and planning activities were carried out in the first phase, including high level meetings with government officials and community mapping for logistic and distribution purposes. Training activities were undertaken in the second phase, and in the last phase (mid-June 2019 to end-July 2019), drug delivery was rolled out to the target population.

### Data collection

All cost data were collected prospectively for the study. Financial reports and supporting documentation were made available by the study coordinator (JLT) on a private and secure online repository six months after MDA implementation. Information concerning human resources, level of involvement and amount of time spent during the MDA was gathered through follow-up interviews with the project nurse coordinator (JM).

### Costing approach

We estimated the cost of MDA in the whole of the Northern Division of Fiji, following the Global Health Cost Consortium (GHCC) reference case. [24] The study used a micro-costing approach, which involves a detailed identification and measurement of the quantity of resources used and respective unit costs and therefore improves the accuracy of the costing exercise.[25] Although labour intensive in terms of collecting detailed information, this approach is useful in estimating the cost of new community-based interventions or new health technologies.[26].

### Categorisation of costs

The cost breakdown was prepared from the perspective of the healthcare payer. The healthcare payer perspective includes treatment costs and other health-related resource utilisation costs associated with the MDA program incurred by a (typically third party) healthcare payer.[27].

In this study, two parties were considered as healthcare payer; the implementing partner (MCRI) and the Government of Fiji. MCRI bore almost all the operational/program costs whilst contributions by the Fijian Government were largely non-financial in nature. Non-program related costs such as research fees, research staff salaries and overseas administration costs were not included in the analysis. We focused our costing analysis on scabies, and costs were allocated proportionately over the number of treatment rounds required for scabies and lymphatic filariasis. Scabies required two treatment rounds whereas the lymphatic filariasis only required one round. Therefore, programmatic costs were divided by three, with two thirds of costs allocated to the scabies program. The analysed data should provide an estimated cost for a scabies MDA program for the Ministry in the future.

The “unit cost per dose administered” was calculated by dividing the total program cost by the number of doses administered to individuals across both MDA rounds. The estimated cost per person was calculated assuming completion of the required two doses of ivermectin by multiplying the unit cost per dose administered by two. A “cost per capita” was determined by dividing the total MDA costs by the total population of the Northern Division derived from the Fiji Bureau of Statistics.[20].

We did not calculate a unit cost “per person treated” as some individuals received only one dose of ivermectin (whether it be in the first treatment round or the second) instead of the required two doses. Data were not available on the number of people who received a full course (two doses) nor those who only received one dose.

Key activities undertaken in the lead up to the MDA and during the course of the intervention were categorised as described previously [28]: 1) advocacy; 2) planning and mapping; 3) mobilization and training; 4) delivery of the intervention drug; and 5) administration activities not otherwise specified (S2 Table).

Using methods defined in previous studies of interventions for NTDs, [29] we categorised costs incurred in each of these five key activity areas according to the type of resource utilised. Types of resources included personnel costs/salaries, per diems, transport/travel, supplies, equipment and other overhead costs necessary for running the MDA campaign. Input costs were classified into financial and economic costs in order to highlight the significant value of donated resources (S3 Table) consistent with the GHCC Reference Case.[24] Financial costs included actual cash expenditures from the perspective of the implementing partner, MCRI. Economic costs encompassed the value of all resources used whether donated or purchased in order to capture the value forgone of all resources used for MDA.[24]. Total costs were converted to US dollars (US$) at the time of the MDA using an average exchange rate (FJ$1 = US$0.4609) as reported on the Reserve Bank of Fiji website.[30] In order to allow comparison of prior cost estimates to our study results, reported costs in the literature were also converted to 2019 US dollars using best practice recommendations.[31].

### Valuation of resources

The MDA was primarily delivered by healthcare workers employed by the MHMS. Their contribution of time during normal working hours was estimated by multiplying individual contractual rates with estimated number of days spent on MDA activities. Nurses were engaged full-time and other health professionals were engaged for an average of 10 days during the 6 weeks of drug delivery. Time contributed by community health workers (CHWs) in delivering ivermectin was assigned a value by apportioning government mandated FJ$200 monthly allowance according to the duration of their involvement in the MDA. With respect to free use of office space and vehicles, market values for rent and hire charges were applied to these resources.

The first dose of ivermectin was donated by the Mectizan Donation Program (MDP) as part of MDA for lymphatic filariasis, [32] and valued as an economic cost. MCRI purchased additional ivermectin based on the forecasted quantity required to treat the target population. Drug costs by MCRI were included in both financial and economic cost estimation. (Table 1). The donated ivermectin tablets by MDP were valued at the same unit price as the negotiated price for the purchase of ivermectin tablets by MCRI with the supplier. All permethrin was purchased by MCRI.

**Table 1:** Valuation of drugs.

Details	Ivermectin		Permethrin
	(3mg tablet)		(30g tube)
	**MDP**	**MCRI**	**MCRI**
	Supply for the treatment of lymphatic filariasis (1^st^ dose)	Purchased by implementing partner (2^nd^ dose)	Alternative treatment for those who did not meet the ivermectin inclusion criteria
Total dosage acquired for MDA	638,072 tablets	620,000 tablets	23,820 tubes
Unit cost per 1,000 tablets/tubes (US$)	$181.75 ^a^	$181.75 ^a^	$1,165.07
Total value (US$)	$114,852	$112,688	$27,752

^a^ MCRI negotiated with the supplier (Laboratorio Elea Phoenix S.A.) for the purchase of 620,000 ivermectin tablets for the price of US$112,688. This equates to a unit cost of US$181.75 per 1000 tablets or US$0.18 per tablet. The MDP donated an additional 638,072 ivermectin tablets as part of a global program to eliminate lymphatic filariasis.

### Sensitivity and scenario analysis

One-way sensitivity analyses were conducted on per diem and overtime costs to explore how a 50% decrease would affect the total and unit cost estimates. This cost category was selected for analysis as it was the largest component of MDA costs and would be unlikely to be as substantial if an MDA program was rolled out by the government alone without the research component included. We also explored an increase in ivermectin drug price of US$0.28 per tablet. In addition to varying per diem and overtime costs and the price of ivermectin we also explored the following scenarios: 1) administration of one dose instead of two doses and 2) non-integrated MDA targeting scabies only. In the scenario where only one treatment round was assumed, total cost in the delivery phase of the MDA were divided by two as most cost items could not be disentangled across the two treatment rounds. For the non-integrated MDA scenario, total training and drug delivery costs were attributed to running the scabies MDA on its own rather than simultaneously with the lymphatic filariasis program. Costs were not shared between the two disease initiatives in this scenario analysis as the same amount of time would have been spent administering the drugs to the population for the treatment of scabies. Investigations into the wholesale unit cost of ivermectin tablets that met accepted international regulatory standard estimated an average price of US$0.28 as communicated by M. Parnaby (Programs Manager, Tropical Diseases Group, Murdoch Children’s Research Institute) in May 2021.

## Results

The MDA campaign in the Northern Division delivered ivermectin to a total of 135,744 and 121,760 individuals during the first and second treatment rounds, respectively. The total financial and economic cost of the campaign across both rounds amounted to US$368,720 and US$627,193 respectively (Table 2). The cost of ivermectin was the largest contributor to the total cost of mass treatment (36.3%), followed by time contributed by MHMS staff (20.5%).

**Table 2:** Costs related to the scabies MDA campaign.

	Total financial costs (US$)	Percentage (%) of financial cost	Total economic costs (US$)	Percentage (%) of economic cost
Permethrin	27,752	7.5%	27,752	4.4%
Fuel & transportation	10,863	2.9%	10,863	1.7%
Office supplies & utilities	16,662	4.5%	16,662	2.7%
Per diem and overtime	124,227	33.7%	124,227	19.8%
Catering & hospitality	5,772	1.6%	5,772	0.9%
Print and media awareness materials	31,416	8.5%	31,416	5.0%
CHW training	7,065	1.9%	7,065	1.1%
Vehicles (rented)	32,275	8.8%	32,275	5.2%
Vehicles (Ministry of Health)	-	0%	7,743	1.2%
Ministry of Health staff time	-	0%	128,209	20.5%
CHW time	-	0%	3,687	0.6%
Ministry of Health office space	-	0%	3,982	0.6%
Ivermectin	112,688	30.6%	227,540	36.3%
**Total**	**$368,720**		**$627,193**	

Using the total number of individuals treated in both rounds, financial and economic unit cost (per dose administered) were estimated at US$1.43 and US$2.44 respectively (Table 3). As data was not available on the numbers of persons who received full treatment, we also estimated intervention unit cost per capita (based on the latest national census). Cost per capita was comparable (US$4.75) to cost per person (US$4.88) as shown in Table 3.

**Table 3:** Mass drug administration unit cost (US$).

Number of persons treated (Rounds 1 and 2)	257,504
Financial cost per dose administered	**$1.43**
Financial cost per person	**$2.86**
Economic cost per dose administered	**$2.44**
Economic cost per person	**$4.88**
Northern Division population *	131,914
Financial cost per capita	**$2.80**
Economic cost per capita	**$4.75**

* Adapted from official census data (Fiji Bureau of Statistics)

Table 4 presents financial and economic costs as a proportion of total cost which are categorized according to activity type. Drug delivery activities incurred the largest economic cost (91.5%), whilst the other categories of activities in the lead up to the drug distribution required less investment.

**Table 4:** Financial and economic costs by activity.

Activities	Total financial cost (US$)	Percentage (%) of financial cost	Total economic cost (US$)	Percentage (%) of economic cost
Advocacy and planning	6,538	1.80%	6,538	1.00%
Mobilisation and training	46,668	12.70%	46,668	7.50%
MDA delivery	315,514	85.50%	573,987	91.50%
**Total**	**368,720**	**100%**	**627,193**	**100%**

### Sensitivity and scenario analysis

**Fig 1:**
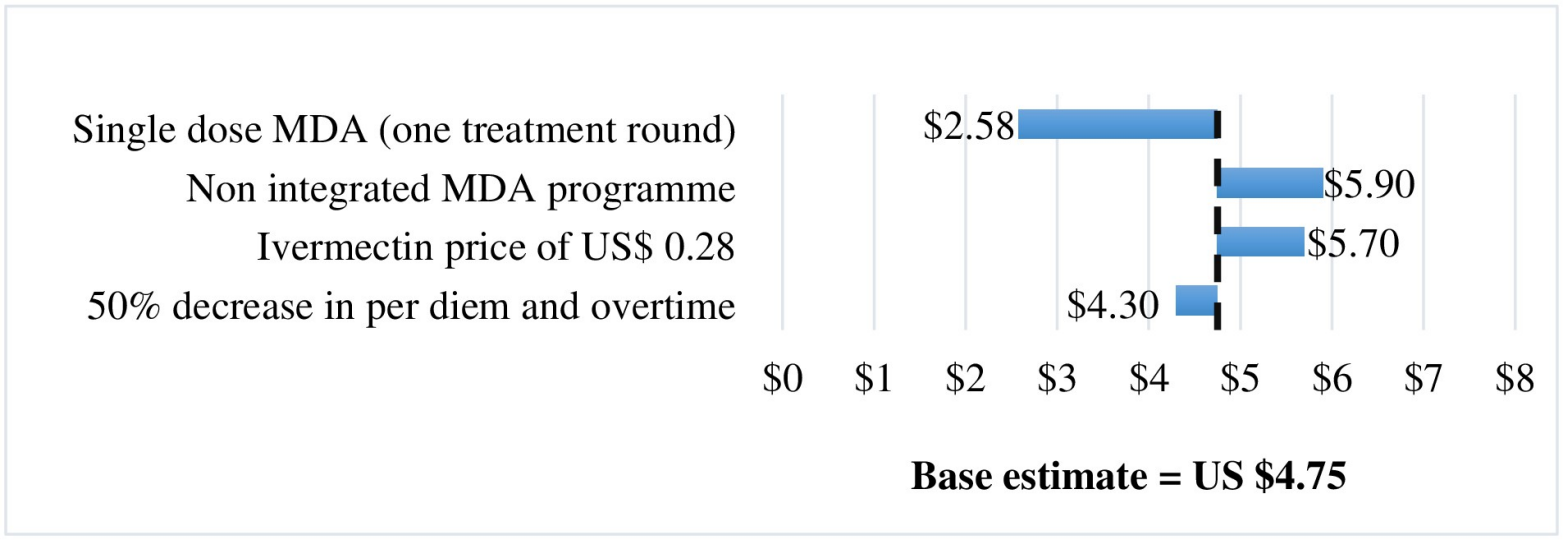
Sensitivity and scenario analysis on economic cost per capita.

The administration of a single dose of ivermectin had the most pronounced impact, (Fig 1) decreasing economic unit cost by 45.7% to US$2.58 (approximating the economic cost of US$2.44 per dose administered). As expected, there was an increase in cost per capita if a non-integrated approach to the MDA was adopted. A similar increase was also observed if the average price of ivermectin tablet was US$0.28 instead of US$0.18. Decreasing per diem and overtime costs had less impact on findings.

## Discussion

This is the first study to establish the cost of administering ivermectin for the control of scabies within an integrated MDA program. The MDA campaign incurred an economic and financial unit cost of US$2.44 and US$1.43 respectively per dose delivered, translating to an estimated economic cost of US$4.88 per person treated with two doses of ivermectin.

These unit costs are higher than those reported for other NTDs where economic cost per person treated has been estimated to be in the order of US$1.00 or less and financial unit costs US$0.50 or less.[16,18,28,33–36] These differences may be attributed to several factors.

First, most MDA costing studies involve a single dose regimen rather than two doses as delivered in this study. Compared to other NTDs such as lymphatic filariasis and onchocerciasis that require only one dose of ivermectin,[37] the current two-dose regimen for scabies contributes to the increased cost estimates. Studies are underway to assess the efficacy of administering a single dose of ivermectin compared to two doses for control of endemic scabies (ACTRN12618001086257).

Second, since this was a first large-scale MDA of its kind to be rolled out in the country in efforts to treat and control scabies, the start-up costs are substantial. The extensive experience in Fiji with national MDA programs for NTD control, including yaws elimination in the 1960s, and the ongoing lymphatic filariasis program,[38] provided the existing infrastructure and ideal context for the scale-up of ivermectin-based MDA for scabies. Inevitably, there was moderate investment in planning activities, mobilising and training health care workers prior to drug delivery. Start-up costs in our study accounted for 8.5% of economic costs, comparable to the 12% estimated in a MDA costing study for trachoma control in South Sudan.[15] In a costing study of MDA for lymphatic filariasis in multiple countries, start-up costs were reported to contribute to high financial and economic costs in initial rounds of MDA, with unit costs decreasing in subsequent rounds.[39] Economic costs per person treated in first rounds of MDA in these countries ranged from US$1.80 to $5.16 (adjusted to 2019 prices: US$3.10 to $8.14). In a 2008–2009 MDA for lymphatic filariasis and soil transmitted helminthiases in Haiti, the financial cost per person treated was estimated at US$0.43 per person treated compared to US$3.10 in the first ever MDA round in 2000.[28] The identification of major cost components in the first year of MDA provides learning opportunities in planning for cost-saving strategies in succeeding years. Furthermore, the findings also highlight important programmatic considerations in designing and implementing similar large-scale MDA in the Pacific such as planned through the WSP.[10].

Third, labour input costs for our study were expected to be comparatively higher than those in previous studies. Fiji is classified as an upper middle-income country by the World Bank, and staff salary scales are higher than in many low-income countries where MDA for NTDs are implemented. Personnel expenditure for staff salaries and overtime payments made up 40.3% (US$252,436) of total economic costs in our study, consistent with other studies where personnel costs have been reported as the main cost driver in other MDA studies, ranging between 37–59%.[15,33,39–41] The research nature and drug delivery component of the program required an all-hands-on-deck approach with a tight schedule of six weeks. Multiple cadres of health care workers were involved and therefore, a considerable amount was expended on salaries, wages, per diem and overtime hours. These likely skewed the cost-estimate towards a ‘worst case’ scenario in this mixed research and health intervention program setting. The significant amounts spent on personnel related expenses were specific to meeting strict research timeline requirements and would not be applicable in budgeting considerations for other large-scale MDA programs such as the WSP. When we performed a sensitivity analysis on per diem and overtime to assess the overall impact on the final cost estimate, we found that an applied 50% decrease would reduce the economic unit cost to US$4.30.

Fourth, the program invested heavily in hiring of third-party and government-owned vehicles in order to achieve high MDA coverage for the 2 treatment rounds. Majority of the Northern Division population reside in isolated rural and maritime communities. The logistical challenges to reach remote communities within the six-week timeframe required additional transportation and financial resources. The results from this study are consistent with WHO findings that unit cost benchmarks have been found to be very sensitive to the scale of MDA implementation and target population density. Furthermore, low-cost estimates for mass drug treatment have been largely derived from costing studies in peri-urban settings.[42].

The value of donated ivermectin represented the second largest proportion of economic cost (18.3%) and its exclusion from the cost analysis would lower the estimated economic cost per person to US$3.98. Many MDA programs focused on NTDs (in LMIC in particular) rely heavily on drug donations, and the financial costs of MDA excluding the value of donated drugs may be more relevant from a healthcare budget perspective, whereas the economic cost including the value of donated drugs is important to assess the cost-effectiveness of MDA programs.[35] The donation of ivermectin by MDP is specifically directed at global efforts for the treatment of lymphatic filariasis and its joint delivery for the treatment of scabies and lymphatic filariasis presents an opportunity for cost savings in subsequent MDA programs. With regard to the pricing of ivermectin tablets, the sensitivity analysis also demonstrated a considerable impact on estimated unit costs. This is in part because the drug prices are heavily influenced by negotiations with the supplier based on quantity ordered and established rapport between supplier and buyer.

Our findings highlight important and unique considerations in the start-up and implementation of a large-scale MDA in the Pacific region and specifically for scabies. The estimated costs of implementing an integrated MDA program for both scabies and lymphatic filariasis can guide stakeholders and policy makers in resource allocation decisions between competing health needs. A majority of activities such as training, community awareness and drug delivery overlapped in this integrated program, representing opportunities for cost-sharing across both disease initiatives. In our study, we estimated total marginal cost of approximately US$146,000 if a scabies MDA program was added to an existing lymphatic filariasis program. Marginal cost is expected to decrease if other disease initiatives are added to the integrated program. In the scenario analysis, the cost per capita in a hypothetical non-integrated program increased by 24% in comparison to running an integrated program. Evans et al.[43] compared costs of converting from separate disease-focused delivery rounds to an integrated MDA for lymphatic filariasis, schistosomiasis, soil transmitted, helminthiasis (STH) and onchocerciasis in Nigeria and found that delivery costs reduced by 41%. As others have shown, an integrated approach, where feasible, can result in cost efficiencies through time savings, higher population coverage and better health outcomes.[44].

### Study limitations

This study has two main limitations. First, placing a value on the time spent by personnel on the MDA was difficult as detailed time records were not readily available. A full-time involvement was estimated for nurses based on interviews with the Big SHIFT study and nurse coordinators, as the arrangement between MCRI and MHMS required full engagement by all nursing staff during MDA delivery. Based on these same interviews, other health professionals contributed an average of 10 days. Second, we were unable to assign costs to either one of the two MDA doses specifically, as the two doses of ivermectin were administered at different time points across the Division, and some cost items were provided in totals across both rounds. Another limitation was the lack of data on the exact number of people who received a full course of treatment (two doses), potentially under-estimating programmatic cost. Unit cost per capita (based on the census) was estimated for comparison purposes with no major variation noted between the two estimates. Despite these limitations, this study provides new evidence on the cost of a large-scale MDA program in Fiji. The study was conducted in Fiji, an upper-middle income country in the Pacific, and so our findings are likely to be fairly representative of the cost of a large-scale MDA program in this region, although less generalizable to low-income countries.

## Conclusion

Our study is the first costing study of MDA for scabies. We found that the costs of MDA for scabies in Fiji were higher than those estimated in previous studies for other NTDs in low-to-middle income countries. Our findings highlight common drivers of MDA costs, attributed to the training and delivery of the MDA program, coverage of hard-to-reach populations, extensive remuneration of health care workers and the required administration of two doses of ivermectin. These particular determinants of cost serve as important references in national health policy decision making in designing and implementing a sustainable and cost-effective MDA program for scabies. Furthermore, the findings from our study will form the basis for a comprehensive cost effectiveness analysis of health and economic benefits derived from the implementation of MDA to guide long-term investment decisions.

## Supporting information

S1 Table Ivermectin dosage regimen. A single oral dose of ivermectin in each treatment round was based on height measurements (per dose pole).
(PDF)

S2 Table MDA key activities. (PDF)

S3 Table Financial and economic costs. (PDF)

S1 Data Full data set. (XLSX)
