# Supplementary material for: Costs of mass drug administration for scabies in Fiji
Source: PLoS Negl Trop Dis. 2022 Feb 3;16(2):e0010147. doi: 10.1371/journal.pntd.0010147 (PMC8846527; doi:10.1371/journal.pntd.0010147)
Supplement: S1 Table — A single oral dose of ivermectin in each treatment round was based on height measurements (per dose pole). (PDF) [file pntd.0010147.s001.pdf]

S1 Table. Ivermectin dosage regimen

Height in centimetres	Single oral dose Number of 3mg ivermectin tablets
90 to 112 cm	1 tablet
113 to 133 cm	2 tablets
134 to 146 cm	3 tablets
147 to 156 cm	4 tablets
157 to 164 cm	5 tablets
165 to 200 cm	6 tablets
