# Supplementary material for: Costs of mass drug administration for scabies in Fiji
Source: PLoS Negl Trop Dis. 2022 Feb 3;16(2):e0010147. doi: 10.1371/journal.pntd.0010147 (PMC8846527; doi:10.1371/journal.pntd.0010147)
Supplement: S2 Table — (PDF) [file pntd.0010147.s002.pdf]

S2 Table. MDA key activities

Activity type	Description
Advocacy	Efforts undertaken to garner support from the Fijian government and other stakeholders for the implementation of MDA. Examples include travel, high level meetings and discussions with government officials.
Planning and mapping	Micro-planning meetings held at the Divisional level, discussions with officers in the various medical areas, finalising population and community profiles, schedules and logistics for MDA.
Mobilisation and training [1]	Activities undertaken just prior to the actual MDA including finalising awareness materials such as pamphlets and media materials, training sessions for health care workers and official visits by the project team to various communities and health facilities to raise awareness.
Drug delivery [2]	Drug distribution and administration of ivermectin/permethrin undertaken by healthcare personnel and community workers.
Administration	Other activities not accounted for in the above categories such as office administration of project resources and office space.

Notes:

1. Training costs were equally apportioned between the scabies and lymphatic filariasis MDA programmes.
2. A single dose is required under the current lymphatic filariasis treatment regimen and we allocated drug delivery costs (in thirds) between the 2 treatment rounds required for scabies and the single round for lymphatic filariasis.
