# Supplementary material for: Costs of mass drug administration for scabies in Fiji
Source: PLoS Negl Trop Dis. 2022 Feb 3;16(2):e0010147. doi: 10.1371/journal.pntd.0010147 (PMC8846527; doi:10.1371/journal.pntd.0010147)
Supplement: S3 Table — (PDF) [file pntd.0010147.s003.pdf]

S3 Table. Financial and economic costs

Input cost component	Included in financial costs	Included in economic costs
Fuel	Yes	Yes
Office supplies	Yes	Yes
Office utilities	Yes	Yes
Communications	Yes	Yes
Per diems	Yes	Yes
Catering for training and meetings	Yes	Yes
Print and media awareness materials	Yes	Yes
Vehicles (rented)	Yes	Yes
Vehicles (Ministry of Health)	No	Yes
Ministry of Health staff time	No	Yes
Community Health Worker (CHW) time	No	Yes
Permethrin	Yes	Yes
Ivermectin	Yes	Yes
Ministry of Health office space	No	Yes
